# Surveys in clinical research: methodological aspects and practical guidance

**DOI:** 10.62675/2965-2774.20260322

**Published:** 2026-04-24

**Authors:** Larissa Bianchini, Aline Braz Pereira, Bruno Martins Tomazini, Cássia Righy, Israel Silva Maia, João Gabriel Rosa Ramos, Regis Goulart Rosa, Roberta Muriel Longo Roepke, Juliana Carvalho Ferreira, Bruno Adler Maccagnan Pinheiro Besen

**Affiliations:** 1 Universidade de São Paulo Faculdade de Medicina Internal Medicine Department São Paulo SP Brazil Postgraduate Program in Medical Sciences, Internal Medicine Department, Faculdade de Medicina, Universidade de São Paulo - São Paulo (SP), Brazil.; 2 HCor-Hospital do Coração Research Institute São Paulo SP Brazil Research Institute, HCor-Hospital do Coração - São Paulo (SP), Brazil.; 3 Universidade de São Paulo Faculdade de Medicina Hospital das Clínicas São Paulo SP Brazil Hospital das Clínicas, Faculdade de Medicina, Universidade de São Paulo - São Paulo (SP) Brazil.; 4 Intensive Care Unit, Centro Hospitalar Unimed Joinville SC Brazil Intensive Care Unit, Centro Hospitalar Unimed - Joinville (SC), Brazil.; 5 Hospital Sírio-Libanês Intensive Care Unit São Paulo SP Brazil Intensive Care Unit, Hospital Sírio-Libanês - São Paulo (SP), Brazil.; 6 Instituto D’Or de Pesquisa e Ensino Rio de Janeiro RJ Brazil Instituto D’Or de Pesquisa e Ensino - Rio de Janeiro (RJ), Brazil.; 7 Instituto Estadual do Cérebro Paulo Niemeyer Rio de Janeiro RJ Brazil Instituto Estadual do Cérebro Paulo Niemeyer - Rio de Janeiro (RJ), Brazil.; 8 Instituto Nacional de Infectologia Fundação Oswaldo Cruz Rio de Janeiro RJ Brazil Instituto Nacional de Infectologia, Fundação Oswaldo Cruz - Rio de Janeiro (RJ), Brazil.; 9 Universidade Federal de Santa Catarina Department of Internal Medicine Florianópolis SC Brazil Department of Internal Medicine, Universidade Federal de Santa Catarina - Florianópolis (SC), Brazil.; 10 Hospital Nereu Ramos Florianópolis SC Brazil Hospital Nereu Ramos - Florianópolis (SC), Brazil.; 11 Clínica Florence Salvador BA Brazil Clínica Florence - Salvador (BA), Brazil.; 12 Universidade Federal da Bahia Faculdade de Medicina Salvador BA Brazil Faculdade de Medicina, Universidade Federal da Bahia - Salvador (BA), Brazil.; 13 Universidade Federal do Rio Grande do Sul Porto Alegre RS Brazil Postgraduate Program in Pneumological Sciences, Universidade Federal do Rio Grande do Sul - Porto Alegre (RS), Brazil.; 14 Hospital Moinhos de Vento Internal Medicine Department Porto Alegre RS Brazil Internal Medicine Department, Hospital Moinhos de Vento - Porto Alegre (RS), Brazil.; 15 A.C. Camargo Cancer Center São Paulo SP Brazil Intensive Care Unit, A.C. Camargo Cancer Center - São Paulo (SP), Brazil.; 16 Universidade de São Paulo Faculdade de Medicina Hospital das Clínicas São Paulo SP Brazil Divisão de Pneumologia, Instituto do Coração, Hospital das Clínicas, Faculdade de Medicina, Universidade de São Paulo - São Paulo (SP), Brazil.; 17 Instituto D’Or de Pesquisa e Ensino São Paulo SP Brazil Instituto D’Or de Pesquisa e Ensino - São Paulo (SP) Brazil.

**Keywords:** Survey, Questionnaire, Survey design

## Abstract

Use of surveys in clinical research allows investigators to explore stakeholders’ perspectives, measure implementation of interventions, and inform future decision-making. Surveys are versatile and accessible, but they require methodological rigor to yield adequate results. Their development involves a sequence of decisions that influence both data quality and interpretability - from defining objectives and selecting a sample to designing the questionnaire and choosing the method of administration. Questionnaires must balance clarity with precision, capturing relevant constructs without overburdening respondents. In many cases, frameworks such as Knowledge, Attitude, and Practice questionnaires are employed to structure questions and explore relationships between what individuals know, believe, and do. Online platforms increase the ability to disseminate surveys to a broader, more diverse target population and to improve data-collection workflows. Beyond the technical aspects, using surveys for clinical research faces practical challenges, such as low response rates and variability in engagement across formats. Addressing these issues requires planning and the use of strategies to encourage participation without compromising data quality. This review offers a practical overview intended to guide researchers in designing and conducting survey studies in clinical research.

## INTRODUCTION

Surveys are used in social science to gather information about participants’ knowledge, beliefs, attitudes, and practices based on a representative sample of the population.^(1)^ In medical research, surveys can be useful from the patient's perspective to understand preferences that impact the care provided, identify disparities, evaluate treatment implementations, inform health policies, and generate hypotheses and feasibility data for clinical trials.^(2–4)^ Many of the challenges faced by surveys in general also apply to surveys in critical care. Even though the number of critical care surveys has increased in recent years, methodological pitfalls, such as frequent convenience sampling, limited participation, and potential discrepancies between self-reported and actual attitudes, persist.^(5,6)^

**Figure 1:**
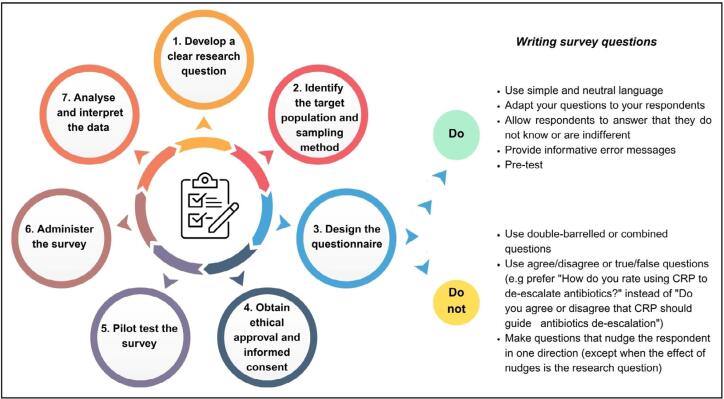
Stages of survey development.

Surveys in clinical research follow a structured sequence of steps to ensure the reliability and validity of the data collected. This process begins with the formulation of clear, focused objectives, followed by the selection of an appropriate sampling frame and the design of a probability-based sample to support valid population-level inferences (Table 1). Next steps include the development of a well-designed questionnaire and a structured data collection process, aiming to obtain adequate response rates (Figure 1).^(4)^

**Table 1:** Do's and dont's of survey research

Do
Know the denominator of your sampling frame to allow the calculation of non-response rates
Avoid confusing or ambiguous questions. You will not be able to analyze and interpret these results later
Remember to consider cultural characteristics and social biases. Avoid social desirability bias
Remember that less is more during item generation and reduction! Do consider using dummy tables to visualize the end product of your survey
Pretest your survey after item generation and reduction. It will save you time and effort in the future
Pilot test your survey. It will avoid deployment of a survey with technical issues
**Don't**
Deploy a survey with unclear research goals. Do define if your goal is to describe, predict or make inferences
Start an online survey if you can't have a clear sampling plan. Sampling errors will lend your results subject to much criticism
Interpret what survey respondents reported to do as an actual observed behavior or attitude. Cognitive biases may influence how humans respond to surveys
Report only interesting findings. Follow your initial research plan to avoid selective reporting
Deploy surveys without ethical approval or consultation of its need with research Ethics Committees

CRP - C-reactive protein.

Overview of the main steps in designing and implementing a survey study in clinical research.

## DESIGN CONSIDERATIONS IN SURVEY RESEARCH

### Defining the research objective

When designing a survey, research objectives should be clearly defined to guide the survey's design. Surveys are useful for understanding knowledge, reported practices, behaviours, attitudes, and the prevalence of specific issues in clinical practice. But surveys can move beyond these issues and use clinical vignettes and even randomization to explore processes and behaviours. They may be meant to describe, predict, or make inferences.^(7)^ Clarifying the survey research question is therefore essential to select the appropriate framework and achieve your goals with the study. Common terms used in surveys in clinical research are explained in table 2.

**Table 2:** A glossary of common terms used in survey research

Term	Definition
Acquiescence	A response bias in which individuals tend to agree with survey statements without carefully considering their content, often leading to inflated correlations
Acronym BRUSO	Acronym highlighting key principles for writing effective survey questions: "brief", "relevant", "unambiguous", "specific", and "objective"
"Ceiling" or "floor" effect	Occur when survey responses cluster at the top ("ceiling") or bottom ("floor") of a scale, thereby reducing data variability
Close-ended questions	Questions that offer respondents a fixed set of answer options to choose from
Construct validity	How well a survey or test measures the theoretical concept or construct it is intended to assess
Content validity	The extent to which a survey or test fully represents the entire domain of the concept being measured
Coverage error	Occurs when some members of the target population are not included in or have no chance of being selected from the survey sampling frame
Criterion validity	How a measure correlates with an external standard or outcome (the criterion), indicating how accurately it predicts or reflects real-world performance
Don't know answer	A response option that allows participants to indicate they have no opinion, are unsure, or lack sufficient information to answer a question
Double-barreled question (combined questions)	A survey item that asks about two or more issues within a single question, making it unclear which part the respondent is answering
Measurement error	The difference between the true value of what is being measured and the value obtained by the survey or instrument, caused by flaws in question design, respondent interpretation, or data collection
Negative worded items	Survey questions phrased with negation or in a negative form, which can be harder for respondents to interpret. They should be avoided, unless they are explicitly part of the research question (e.g., to evaluate framing effects)
Nonresponse error	When individuals selected for a survey do not participate or fail to answer certain questions, the results can be biased if the nonrespondents differ systematically from respondents
Open form question	Allows respondents to answer in their own words, providing detailed information but requiring more effort to analyze and code, usually with qualitative synthesis methods
Pilot test	Before administering a survey, a pilot test with a small sample of the target population is necessary to identify any issues that need to be corrected before deployment. This includes the cover page, duration, formatting and any other technical issues
Pretesting the questionnaire	Pretesting the survey occurs before pilot testing. During pre-testing, the other authors of the group or close colleagues should read the survey attentively, looking for questionnaire wording and formatting that may lead to incorrect answers. This includes but is not limited to possible induction of social desirability bias, double-barreled questions, acquiescence and possible ceiling and floor effects
Recall bias	A type of measurement error that occurs when respondents do not accurately remember past events or experiences
Sampling bias	Occurs when the sample selected for a study is not representative of the target population
Social desirability bias	Occurs when respondents answer questions in a way they believe is more socially acceptable or favorable, rather than being truthful, which can distort survey results. This may be especially true for sensitive issues that the survey investigator needs to be aware of

### Selecting the survey framework

#### Role of randomization

Randomization in surveys is used to establish causal inference by assigning respondents to different experimental conditions in a way that minimizes bias. In survey experiments, participants are randomly assigned to different groups, allowing researchers to manipulate specific variables - such as question wording, framing, or order - while holding all other factors constant. This process creates equivalent groups at baseline, and any observed differences in responses can be attributed to the experimental manipulation.^(8)^ An example is the study investigating whether different information frames and number formats influence patients’ perception of health risk. Survey participants were randomly assigned to receive risk information in a negative, positive, or combined frame and in frequency or percentage format. The authors found that a negative frame and a frequency format increased risk perceptions for less numerate respondents.^(9)^

#### Knowledge, Attitudes, and Practices questionnaires

Knowledge, Attitude, and Practice (KAP) surveys are used in public health to assess what people know, believe, and do regarding health topics. Knowledge, Attitude, and Practice are a useful framework to design surveys, as they allow for the assessment of the effectiveness of health interventions and education programs over time.^(10)^ A challenge is to design questions that align with the research objectives and capture the three desired aspects, as they may not account for cultural nuances, contextual factors, and reasons behind certain practices.^(11)^ In addition, it is important to acknowledge that what research participants report they believe and do may not reflect what they actually believe and do in practice. This is known as social desirability bias, meaning participants may be inclined to respond in ways they think are more acceptable to the researchers. Knowledge, Attitude, and Practice surveys should be reported according to the ChecKAP checklist.^(12)^

#### Clinical vignettes

Clinical vignettes may also be used in surveys to assess respondents’ perceptions and attitudes.^(2,13–19)^ Vignettes are easy to use, cost little, and allow quantification of respondents’ performance.^(20)^ Moreover, vignettes can be subject to randomization, which allows for the assessment of the impact of specific characteristics on the responses.^(13,16,17,19)^ Even though most evidence supporting the validity of vignettes for assessing clinicians’ performance comes from low-risk conditions, vignettes have been used to study clinicians’ attitudes in acute, time-sensitive settings.^(2,15–17)^ Nevertheless, the vignette methodology may be less robust than encounters with real patients. This is because it summarizes and standardizes the clinical information, is based on static descriptions, and therefore is subject to different interpretations.^(20)^ Those limitations may be mitigated by the utilization of vignettes based on real patients and the utilization of electronic vignettes.^(14,16,20)^

### Determining the sampling method

Sampling determines who will be included in the survey and assures that the collection of information from a subgroup of individuals will be representative of the population of interest.^(21)^ Simple random, stratified, or cluster sampling are options of sampling designs.^(22)^ The target population of the survey is the group of individuals about whom information is desired and to whom inferences are generalized through the survey sample. The inclusion and exclusion criteria of the sample directly reflect the target population.^(21)^ There are two main types of sampling designs:

1. Probability sampling designs: assume that each element in the population has a non-zero chance of selection; they use confidence levels and error margins to estimate the true population effect. Types of probability sampling are described in table 3.^(21,23,24)^
2. Non-probability sampling designs: any sample that does not qualify as a probability sample; used for in-depth investigations of groups that are difficult or expensive to reach, where probability sampling methods are largely inaccessible or unfeasible, including cases that may not be fully representative of the population from a scientific standpoint.

**Table 3:** Methods of probability sampling

Design	Definition	Methods	Advantages	Disadvantages
Simple random sample	Each element in the population has a known, equal, and non-zero chance of being included in the sample	Random number selection or drawing lots	Requires little prior knowledge about the studied population	May not capture certain groups of interest. Might be inefficient
Systematic random sample	Similar to simple random sampling The researcher selects a random starting point and, at a specified interval, systematically selects cases from a sampling list	Systematic selection of cases using a fixed interval from a random start point	Easy to analyze data and compute sampling errors. High precision	Periodic ordering in the sampling list may introduce bias. May not capture certain groups. Might be inefficient
Stratified sample	Used when there is a particular interest in ensuring that certain groups will be included in the study or sampled at a higher (or lower) rate than others	The sample is divided into homogeneous subgroups (strata), and simple or systematic random sampling is used within each stratum	Captures key subgroups of interest. Allows disproportionate sampling and optimal allocation within strata. Maximum precision	Requires prior knowledge of the population. Increases complexity of data analysis and error computation
Cluster sample	Aims to maximize sample dispersion across the community to fully represent existing diversity while minimizing costs	The sample is divided into heterogeneous groups/clusters (e.g., people in certain geographic areas), and a random sample is drawn from the clusters	Reduces fieldwork costs. Allows sampling of groups for which individual-level data may not be available	Greater complexity in data analysis and error computation. Lower precision

Source: adapted from: Aday LA, Cornelius LJ. Designing and conducting health surveys. A comprehensive guide. 3rd ed. San Francisco: John Wiley & Sons; 2006.^(21)^

Several published surveys in intensive care use non-probabilistic sampling designs, often relying on convenience sampling.^(25,26)^ Non-probability sampling methods include:^(21,23,24)^

- Purposive sampling*:* Individuals are selected based on the researcher's judgment because they meet specific criteria:
  – Quota sampling*:* aims to obtain a specific number of respondents with pre-specified characteristics so that the total sample has the same distribution of assumed traits present in the population under study.
  – Convenience (block) sampling*:* individuals are selected based on their random availability at the time of the study.
  – Snowball sampling*:* investigators identify one or a few participants who are representative of a difficult-to-locate population of interest, who, in turn, identify other potential respondents who meet the same criteria.^(21,23)^

When designing a sampling strategy, one must carefully assess precision, accuracy, complexity and efficiency. Precision is how close the estimates derived from a sample are to the true population value as a function of variable sampling error. Accuracy refers to how close the estimates are to the true population value as a function of systematic error or bias. Complexity reflects the amount of information needed prior to the study and the number of stages and steps required for implementation. Efficiency means obtaining the most precise estimate at the lowest possible cost, and the match between the sample design and the research objectives.^(21)^

#### Sampling error

**Figure 2:**
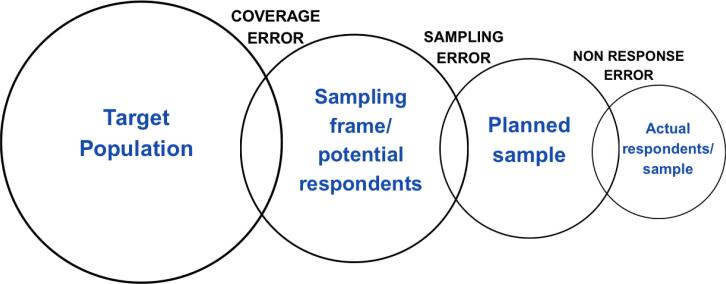
Sampling error framework

The process of estimating characteristics of a target population based on a sample of respondents may introduce errors, which can be classified into two main types: random error and systematic error (or bias). These errors arise at different stages of the sampling process and can affect the selection of respondents and the accuracy of their responses.^(27)^ Random error originates from the fact that data are collected from a subset rather than the entire population. Systematic sampling errors occur when the sample consistently over- or underrepresents certain segments of the population. Three main sources contribute to this bias: (1) coverage error, when the sampling frame does not fully cover the target population; (2) sampling bias, if the method of selecting individuals from the sampling frame is not random, as relying on volunteers or convenience samples; (3) nonresponse error, when not all invited individuals participate, and the nonrespondents systematically differ in meaningful ways from respondents (Figure 2). Beyond selection bias, other systematic errors include measurement errors due to misunderstanding questions, recall bias, or social desirability bias.^(28)^

Sampling frame phases from the target population to actual respondents, describing potential sources of error: coverage error, sampling error, and non-response error. We propose a survey targeting Brazilian board-certified intensivists (the target population). Potential respondents are intensivists registered with the *Associação de Medicina Intensiva Brasileira* (AMIB), but not all of them may have a current AMIB registry, leading to coverage error when using this sampling frame. From this frame, you plan to sample 10-20%, but some respondents are not board-certified intensivists but rather physicians currently working in the intensive care setting and part of AMIB. This could lead to sampling error. Finally, not all respondents in the planned sample may complete the survey, which could lead to non-response error if non-respondents have different characteristics or respond differently to the survey than respondents.

#### Sample size calculation

To calculate a simple random sample for a survey, researchers must define the target population and the variables of interest. Calculation also depends on the aim of the survey. If the goal is descriptive, the focus is on estimating a population proportion, mean, or other parameter. If it is an analytical study, groups will be compared, and standard sample-size calculations are required.^(29)^ For example, to use a proportion, researchers need to estimate the expected proportion (p) of the population that has the characteristic being studied, then choose a confidence level (commonly 95%) and a margin of error (for example, ± 5%) to conduct the sample size calculation.^(30)^ We provided the sample size formulas for a mean and for a proportion, with an example of sample calculation in the Supplementary Material.

The calculated simple random sample serves as the baseline and is adjusted by a design effect when more complex designs are employed. Stratified sampling typically reduces the required sample size by increasing precision, whereas clustering and the use of weights tend to increase it. Multistage sampling often combines these different techniques and balances their effects.^(31)^

## METHODOLOGICAL ASPECTS IN QUESTIONNAIRE DEVELOPMENT

Development of a robust questionnaire involves implementing at least six methodological steps, listed as follows: item generation, item reduction, questionnaire formatting, questionnaire composition, pre-testing and piloting, and validation.

### Item generation

Choosing a well-defined question is the first step to developing a concise questionnaire, avoiding long instruments that exhaust the respondents and reduce response rates.^(32)^

### Item reduction

After the initial version of the questionnaire is completed, authors reanalyze it aiming to identify repeated domains or redundant questions. Usually, up to five main thematic areas are recommended, as more domains may tire the respondent and reduce survey completion. Reduction occurs through a similar process of item generation: evaluation by investigators, other experts, participant feedback, focus groups, or statistical analysis of item correlations within a domain.^(35,36)^ Despite the importance of an appropriate methodology report, a review showed that most medical surveys lack descriptions of the survey design: domains were reported in 59.1% of the studies, item generation in 33.1%, and item reduction in 12.6%.^(5)^

### Questionnaire formatting

Stems and the format of the response items may affect response rates. Stems must remain simple and cover only one variable at a time. Besides, questions should be short, limited to 12 to 20 words.^(33,37)^ The use of terminology perceived as biased or judgmental affects respondents, and special attention to neutral terms is necessary when a question concerns respondents’ values. Terms such as "usually", negatively worded items, combined questions in the same item (also called double-barreled questions), vague questions, unfamiliar terms, and overlapping answer choices should be avoided.^(38)^

Response options range from direct, closed-ended formats with a limited number of choices to open-ended formats. Open-format responses can provide insight into a topic, but summarizing them becomes difficult with larger samples and requires a specific qualitative methodology to be analyzed.^(29)^ Using an "other" option in closed-loop questions is an alternative to allow different answers from the initially anticipated. Closed-loop answers measure the degree of certain constructs through a scale. Likert-type scales, rating scales, pictorial scales, visual analog scales, rank lists, and semantic differential scales are some of the response formats available to adopt.

Likert-type scales have answers in various formats, including agreement (e.g., "strongly disagree" to "strongly agree"), frequency (e.g., "never" to "always"), extent, and similarity.^(39)^ Likert responses follow a normal distribution and present a small risk of bias when applied in large and methodologically well-designed surveys.^(40)^ However, they may still hold the risk of acquiescence when compared to item-specific options. Acquiescence means that individuals may tend to agree with different survey items indiscriminately, leading to arbitrary correlations between unrelated questions.^(41)^

Other scales use different indicators to measure responses. Examples include a score from one to ten in rating scales; images to represent feelings in pictorial scales; continuous lines where respondents select a point that reflects the intensity of the evaluated measure in visual analog scales; and pairs of opposite adjectives as "useful" *versus* "useless" in semantic differential scales.^(42)^

Numerous scale points may tire the participants and generate either a "ceiling" or "floor" effect. In this scenario, respondents choose answers closer to the top or the bottom of a scale, and true response variability may not be captured.^(43)^ In KAP surveys, it is advisable to include a "Don't know" answer, as it reduces the chance of the question not being answered and avoids respondents choosing an option that they disagree with.^(44)^

### Questionnaire composition

Before the initiation of the questionnaire, a cover letter is included to explain the purpose and the impact of the survey and to provide the estimated time needed for participants to complete it. Demographic questions are usually placed in the beginning of the questionnaire except if they are related to more sensitive topics - in which case, they should be asked towards the end. The questionnaire is displayed in a logical order with questions on the same topic clustered. Broader questions can be placed at the beginning, and they gradually progress to specific ones or more important questions can be asked first when fatigue is less likely. Introductory or summary questions improve the flow of the questions. Using adequate size and color combinations for the survey is also important - a study found that a large questionnaire printed on blue paper produced better response rates.^(45)^ Questionnaire length presents a bigger impact on answer accuracy than on response rate.^(37)^

### Pre-testing and pilot testing

After item generation, reduction, questionnaire formatting, and composition, the survey should be pretested by content experts (authors or non-authors of the survey) who were not involved in the questionnaire design. During pre-testing, one should be attentive to questionnaire wording and formatting that may lead to incorrect answers. This includes, but is not limited to, double-barreled questions, acquiescence, and possible ceiling and floor effects (Table 2).

Pilot testing evaluates the questionnaire's application to a small sample of the target population before the formal launch. It helps identifing ambiguous phrasing, technical glitches, problematic skip-logic paths, cultural mismatches, and other issues that may compromise data quality.^(46)^ Open-ended feedback fields during the pilot phase allow respondents to suggest improvements. Robust pilot testing saves time and resources by reducing the risk of poor data quality or response bias in the full-scale study.^(32,46)^ During pilot testing, authors should also assess the duration required to complete the questionnaire, which is useful to evaluate feasibility. Additionally, pilot testing offers a chance to define success thresholds, such as item non-response rates or completion rates, that can inform instrument refinement.

### Validation

Specific questionnaires and scales for widespread use need to follow a validation framework (e.g., the 36-Item Short Form Health Survey, the Rankin scale). These are usually not reported in surveys designed to answer a single, specific research question, although concepts of validation may help develop a robust survey.

The classical validation framework comprises "validity" and "reliability".^(47)^ Validity is defined as "the degree to which evidence and theory support the interpretation of scores or questionnaires entailed by proposed uses".^(48)^ It is divided into three different subsets: (1)"content validity", which refers to the fact that survey items must constitute a relevant and representative sample of the domain being measured, usually evidenced by the existence of procedures for item development and sampling; (2) "criterion validity", which refers to the correlation between the survey responses and some "criterion", or hypothetical truth, and is evidenced by the correlation with a gold standard; (3) "construct validity", which refers to the variation of responses based on a underlying construct, which is usually evidenced by the correlation with another measure of the same construct, factor analysis or the expected changes in responses between subgroups. Factor analysis is used to investigate relationships between the items in an instrument and the construct by clustering items as the underlying factor structure. Principal component analysis is used for the same purpose but determines whether the survey can have items removed without sacrificing measurement quality.^(47,48)^

Reliability refers to the "reproducibility or consistence of scores across two or more observations that intend to measure the same construct".^(48)^ Reliability may be assessed as internal consistency (reliability across items), inter-rater reliability (across different respondents) or test-retest reliability (across the same test administered in different time periods). Reliability may also be assessed between different platforms, such as when a survey originally developed for in-person interviews may be used in online questionnaires. Reliability can be reported as a coefficient, such as Cronbach's alpha, which assesses the reliability of multiple items across many observations. Kappa's values, alternatively, may be used to assess the reliability of a single observation or to compare inter-rater reliability across a group of raters.^(48)^

## SURVEY APPLICATION CONSIDERATIONS

The selection of data collection methods, such as online or phone surveys, determines both the types of data collected and the number of participants who respond. Reasonable response rates are essential for drawing valid surveys conclusions.

### Modes of administration

Surveys may be conducted through interviews or questionnaires (in person, by mail, by e-mail, or online).^(49)^ In all modalities of surveys, it is important to consider the clarity of questions and the length of time to complete the survey.^(49,50)^

Phone interviews may be less self-selective and have a higher completion rate.^(51)^ In-person interviews tend to have higher response rates but are more expensive and time-consuming. Self-completion questionnaires are cost-effective compared to other methods. Postal or internet surveys can reach widely dispersed populations at minimal cost.^(50)^ Internet-based surveys can be administered by e-mail, by posting to newsgroups, and on the Web using fill-in forms.^(52)^ The public survey link is the simplest and fastest way to anonymously collect responses, and the use of a participant list allows the researcher to send a customized email to potential participants and track respondents. Electronic distribution of cover letters via email attachments or embedded links, combined with digital incentives such as coupons and prize draws, can enhance survey participation, but may also yield biased results.^(53)^ However, online surveys present limitations: potentially unbalanced respondent profiles, since online participants are generally younger, more educated, and less ethnically diverse than the general population; there is an increased likelihood of duplicate responses; and limited control over participants’ surrounding environment or distractions during completion.^(54)^

Links to online surveys and requests to participate in them are now also a common sight in WhatsApp groups. However, this approach raises methodological concerns, including unclear and non-representative sampling, inability to calculate response rates, and lack of information on nonresponders. As a result, findings from such surveys are generally limited in generalizability and are better suited to generating hypotheses or piloting instruments than to drawing inferential conclusions.^(55)^

It is recommended that even online surveys use a defined sampling frame from which participants are randomly selected, as we illustrated in figure 2, to reduce selection bias. Duplicated responses can be minimized by directly identifying the respondents or by using access tracking to detect repeated entries. Notably, online surveys can only be conducted if the target population has reliable internet access. In addition, collecting sociodemographic data enables survey weighting, reducing demographic imbalances in interpreting survey results.^(32,52)^

SurveyMonkey,^(56)^ QuestionPro®^(57)^ and institutional platforms provide researchers with tools to design questionnaires and collect data. Google Forms is widely available and enables real-time data sharing and response tracking via e-mail addresses, though it lacks advanced features such as automated branching. Another option is Research Electronic Data Capture (REDCap), a secure, web-based application designed to support data collection for research studies. It allows researchers to build and deploy customizable surveys using a user-friendly interface, with features like branching logic, real-time data validation, multi-language support, and automated invitations/reminders. Importantly, we strongly suggest using data validation to avoid, by design, wrong or uninterpretable responses. Surveys created in REDCap can be distributed via public or personalized links, and responses are stored in a centralized database with audit trails.^(58,59)^ Although REDCap is free for academic and healthcare institutions’ use, access is limited to institutions that have a formal agreement with the REDCap Consortium, which can be a barrier for researchers whose institutions are not affiliated.

### Response rates

The mode of survey administration (in-person, telefone or mail) can introduce important specific biases related to participant demographics and item nonresponse.^(50,60,61)^ Mixed-mode approaches are likely to yield the highest overall response rates.^(50,60)^

Although there is no single definition of an adequate response rate, a minimum rate of 70% is considered ideal.^(1,62)^ However, in practice, such rates are difficult to achieve, especially in web-based or e-mail surveys, where response rates commonly fall below 50%. After sending the questionnaire, each follow-up mailing yields 30% - 50% of the number of initial responses.^(63)^ Sending up to two reminders appears to be ideal for improving web survey response rates, with the first reminder typically having the greatest impact. The optimal timing for the initial reminder was approximately 3 days after the survey invitation.^(64)^

The use of monetary or non-monetary incentives leads to better survey participation, but it also determines which participants will respond and creates additional bias, as previously mentioned.^(65)^ Other suggestions for generating a good response rate include: contacting participants before sending the questionnaire, promoting the survey, communicating to respondents how their answers contribute to the research, assuring confidentiality, and sharing the survey results with respondents.^(50,66–70)^

## INTERPRETING AND REPORTING SURVEYS

### Handling incomplete data

Incomplete data in surveys can be a consequence of sampling error or actual item non-response. Coverage and non-response errors are addressed through survey weighting, as previously described in the text. Item non-response, however, requires additional considerations. In this scenario, missing data may occur completely at random (MCAR), when missing occurs by chance, unrelated to any characteristic of the participants; at random (MAR), when the probability of missingness depends on information observed in the dataset (e.g., junior physicians being more likely to complete a survey than senior physicans, making missingness related to a known training level); or not at random (MNAR) when incomplete data depends on the unobserved value itself (e.g., as overburdened ICU staff choosing not to report their job satisfaction).^(71)^ The decision of what is the main mechanism of missingness influences how the data will be handled and is ultimately a judgment call by the researchers. Ideally, this should be anticipated and researchers should pre-specify how potential item non-response will be managed. Ignoring missing data implicitly assumes that data are missing completely at random, which is often an oversimplified assumption in practice. Complete case analysis is only reasonable if missingness is mainly MCAR. Multiple imputation is the best valid approach when missingness is MAR. When data are MNAR, there is no consensus on the optimal approach, but including a sensitivity analysis is an option.^(72)^

### Key considerations

Although surveys can help answer many questions, there is considerable heterogeneity in their conduct and reporting, which may compromise interpretation.^(4,73)^ Concerns have been raised about the published surveys’ methodological quality and that the conclusions of surveys may not consider important biases.^(4,74,75)^ As such, both investigators conducting surveys and informed readers trying to make sense of published results could benefit from a systematic approach to the conduct and reporting of surveys.

Guidelines for reporting surveys, such as SURGE and CHERRIES, have been recommended, but both have limitations.^(76)^ More recently, a consensus-based checklist for reporting of survey studies (CROSS) has been developed following the Enhancing the QUAlity and Transparency Of health Research (EQUATOR) directives and is proposed as an alternative to previous guidelines.^(76)^ CROSS was developed systematically and proposes a structured checklist as a guideline for reporting survey results. The CROSS checklist is comprehensive and may help both investigators and readers deal with this problem.

Overall, surveys must report^(13)^ the rationale for the survey, including specific objectives;^(2)^ the methodology, including the creation or adaptation of the instrument, pretest and validation of the instrument, ideally providing the instrument (so that readers can interpret the understandability of the instrument), the method of administration of the instrument and data collection, sampling techniques and sample size calculation.^(14)^ The authors should also describe the results, including response rate, characteristics of the respondents, and evidence of reliability and validity;^(15)^ discussion of the results, including scope of conclusions, study limitations, and generalizability of the results. Moreover, one should identify authorship, potential conflicts of interest, and the role of funding sources.^(4,74,76)^

When presenting and discussing study results, especially in KAP-like surveys, the authors must interpret the results as reported behaviours, attitudes, and practices rather than as actual observed practices.^(77)^ This may be an important limitation when interpreting survey study results, depending on the research question.

## ETHICAL ASPECTS

Surveys must adhere to ethical and regulatory standards, including prior approval by a Research Ethics Committee (REC). Researchers are responsible for ensuring informed consent, voluntary participation, clear disclosure of potential risks and benefits, and strict confidentiality of data.

Effective data protection strategies are essential and may include anonymization, restricted access, secure storage, and transparency in data handling procedures. Researchers are also responsible for using validated instruments, minimizing bias, and ensuring transparent dissemination of findings. Moreover, ethical considerations also extend to data sharing and long-term use of the information collected, which should comply with data protection regulations such as the General Data Protection Regulation (GDPR) in the European Union, or the *Lei Geral de Proteção de Dados* (LGPD) in Brazil.^(78)^ Notably, while ethical approval through a REC and informed consent are usually required, not all survey studies mandate formal REC approval, depending on the nature of the data and institutional guidelines. Researchers are encouraged to consult their local ethics committees to determine the requirements for their specific project.

## CONCLUSION

Survey research is achievable and a powerful approach for generating empirical insights in clinical settings. Nonetheless, proper design, sample selection, and questionnaire development are essential for adequate implementation. Advances in digital platforms over the past few years have expanded access to online survey tools, even as non-response rates remain a major challenge. We hope this review provides researchers with tools to interpret published surveys and develop high-quality surveys for their own research projects.

## Data Availability

The contents underlying the research text are included in the manuscript.
