# Supplementary material for: Surveys in clinical research: methodological aspects and practical guidance
Source: Crit Care Sci. 2026 Apr 24;38:e20260322. doi: 10.62675/2965-2774.20260322 (PMC13155776; doi:10.62675/2965-2774.20260322)
Supplement: Supplementary file 1 [file 2965-2774-ccsci-38-e20260322-suppl01.pdf]

Critical Care Science
https://doi.org/10.62675/2965-2774.20260322

SUPPLEMENTARY MATERIAL

Surveys in clinical research: methodological aspects and practical guidance

Larissa Bianchini[1,2,3], Aline Braz Pereira[4], Bruno Martins Tomazini[2,5], Cássia Righy[6,7,8], Israel Silva Maia[2,9,10], João Gabriel Rosa Ramos[11,12], Regis Goulart Rosa[13,14], Roberta Muriel Longo Roepke[3], Juliana Carvalho Ferreira[15,16], Bruno Adler Maccagnan Pinheiro Besen[1,15,17] on behalf the BRICNet*

* Brazilian Research in Intensive Care Network

Table 1S - Sample size calculation

Steps<br><br>1. Define: purpose of the sample, necessary degree of precision, potential consequences of the findings, acceptable error	For a mean (absolute error)<br><br>$n = \frac{z^2 \cdot S^2}{e^2}$
2. Find the equation that connects the sample size to your goal	For a proportion<br><br>$n = \frac{z^2 \cdot p(1-p)}{e^2}$
3. Estimate unknows values (consider a pilot sample if there is no information available)<br><br>4. Reassess if the sample is too large	Finite population correction<br><br>$n^{adj} = \frac{n0}{1 + \frac{n0 - 1}{N}}$

N is the required sample size, z is the z-score for the desired confidence level, *S* is the estimated standard deviation, *e* is the acceptable margin of error, *p* is the expected population proportion, *N* is the population size, $\bar{x}$ is the estimated population mean, *r* is the tolerable relative error, and *no* is the initial sample size calculated before applying the finite population correction.

This is an open access article under the CC BY license (https://creativecommons.org/licenses/by/4.0/).

FINITE POPULATION CORRECTION

When the total population size (N) is known and relatively small, the finite population correction adjusts the sample size calculated for an "infinite" population to avoid oversampling. This adjustment reduces the required sample size because sampling a large fraction of a small population yields more information than in an infinite population.

PRACTICAL EXAMPLE

Suppose you are designing a survey on the use of procalcitonin to de-escalate antibiotics in patients with suspected infection. You want to estimate how many clinicians use this strategy, with a ± 5% margin of error and 95% confidence (z-score from the normal distribution = 1.96).

Since you do not have prior data, you assume the most conservative estimate, in which 50% of clinicians use procalcitonin for de-escalation. Using the formula for estimating a proportion, you calculate:

$$n = \frac{1.96^2 \cdot 0.5\,(1-0.5)}{0.5^2} \approx 385$$

This means that at least 385 individuals are needed to estimate the proportion with the desired precision.

If you expect, based on prior knowledge, that only 30% of clinicians use procalcitonin for this purpose, the required sample size decreases to about 323 respondents.

However, if the total number of clinicians eligible for the survey – the sampling frame - is known and relatively small, for example, n = 500, you can apply the finite population correction to adjust the sample size:

$$n^{adj} = \frac{385}{1 + \frac{385-1}{500}} \approx 218$$

Thus, after applying the finite population correction for a sampling frame of 500 clinicians, the required sample size decreased from 385 to 218 individuals.

Now, suppose your goal is to estimate the average number of days clinicians wait before de-escalating antibiotics guided by procalcitonin in patients with suspected infection. Based on prior studies or a pilot study, you estimate this average to be about 7 days, with a standard deviation of 3 days. You want to estimate this mean with a margin of error of ±1 day at 95% confidence (z-score from normal distribution = 1.96).

Using the formula estimating sample size using a mean:

$$n = \frac{1.96^2 \cdot 3^2}{1^2} \approx 35$$

This means that at least 35 respondents are needed to estimate the average waiting time with the desired precision.
